# Dalfampridine for Mobility Limitations in People With Multiple Sclerosis May Be Augmented by Physical Therapy: A Non-randomized Two-Group Proof-of-Concept Pilot Study

**DOI:** 10.3389/fresc.2021.795306

**Published:** 2022-01-11

**Authors:** Prudence Plummer, Silva Markovic-Plese, Barbara Giesser

**Affiliations:** ^1^Department of Physical Therapy, MGH Institute of Health Professions, Boston, MA, United States; ^2^Department of Neurology, Thomas Jefferson University, Philadelphia, PA, United States; ^3^Multiple Sclerosis Neurology, Brain Health Center, Pacific Neuroscience Institute, Santa Monica, CA, United States

**Keywords:** physical therapy, rehabilitation, dalfampridine, gait, walking, multiple sclerosis

## Abstract

**Purpose:** To demonstrate proof-of-concept for a combined physical therapy and pharmacological intervention and obtain preliminary estimates of the therapeutic efficacy of a motor-relearning physical therapy intervention with and without concurrent dalfampridine treatment on gait speed in people with mobility limitations due to multiple sclerosis (MS).

**Methods:** Using a non-randomized, two-group design, 4 individuals with MS newly prescribed dalfampridine as part of their routine medical care, and 4 individuals with MS not taking dalfampridine completed a 3-week drug run-in or no-treatment baseline, respectively. After 3 weeks, all participants commenced physical therapy twice weekly for 6 weeks. Participants taking dalfampridine took the medication for the study duration. The physical therapy program comprised functional strengthening, gait training, balance training, and dual-task training. The primary outcome was Timed 25-foot Walk (T25FW) at the end of the 6-week physical therapy program.

**Results:** For the 4 participants taking dalfampridine, average improvement in T25FW on drug only was 12.8% (95% CI 1.2 to 24.4%). During the 6-week physical therapy phase, both groups significantly improved T25FW, but the effect tended to favor the group taking dalfampridine (mean difference = −0.93 s, 95% CI −1.9 to 0.07 s, *p* = 0.064, *d* = 1.6). Whereas the physical therapy group had average T25FW improvement of 10.8% (95% CI 1.0 to 20.5%), the physical therapy plus dalfampridine group demonstrated average improvement of 20.7% (95% CI 3.8 to 37.6%).

**Conclusions:** Further research is warranted to examine whether dalfampridine for mobility impairment may be augmented by physical therapy in people with MS.

## Introduction

Clinical efficacy of oral dalfampridine extended-release for improving gait speed in people with multiple sclerosis (MS) has been demonstrated in two Phase 3 clinical trials (1, 2); however, only 38% of patients taking dalfampridine were “responders” to the medication (3). Responders were defined as patients whose Timed 25-Foot Walk (T25FW) gait speed was faster for at least 3 of the 4 on-drug assessments compared to their fastest off-drug assessment. While the average T25FW improvement of 25% observed in responders is impressive (3), equivalent to 0.16 m/s (4), there are a large number of patients who do not achieve a meaningful response to the medication and for whom adjunct or alternate interventions for gait impairment are necessary.

Task-specific gait training (5–8) such as that provided in physical therapy can also produce positive effects on gait speed in people with MS (4). Although the precision of effect size estimates for gait training are currently limited by very small rehabilitation studies (4), interventions with promising effect sizes have included conventional gait training (6) as well as robot-assisted and treadmill-based gait training (5, 9, 10). Further, it is possible that combining physical therapy with dalfampridine may increase the effect size of dalfampridine on gait speed and the proportion of patients who experience a clinically meaningful treatment effect (11).

The purpose of this pilot study was to demonstrate proof-of-concept and obtain preliminary effect size estimates of dalfampridine combined with physical therapy (D+PT) after an initial drug-only run-in phase to determine responsiveness to dalfampridine alone, and to compare the effects to physical therapy without dalfampridine (PT) in people with MS, on gait speed assessed under fastest comfortable (i.e., T25FW).

## Methods

This pilot study was a non-randomized two-group design with pre-post assessment and 1-month follow up. To be included, participants had to have a diagnosis of MS (any phenotype) and either have been prescribed dalfampridine by their neurologist as part of their usual care or not taking dalfampridine (and not have previously taken it). Participants in both groups had self-reported issues with mobility and/or falls, were 18–70 years old, could complete the T25FW in 6–45 s without physical assistance, and were able to follow a 3-step verbal command in English. Individuals were not eligible if they had experienced an exacerbation in the last 60 days, recent myocardial infarction or illness requiring hospitalization, reported a history of any other neurological disease, lower extremity amputation, or uncorrected hearing impairment that would prevent ability to perform the dual-task assessment. All participants provided written informed consent. The study was approved by the local Institutional Review Board.

Twelve participants were enrolled and 8 participants completed all study related interventions and primary outcome analyses. The 4 withdrawals (non-completers) were related to inability to complete study visits according to schedule (*n* = 1), development of medical issues that prevented continuation in the study (*n* = 1), or substantial delays in obtaining approval from insurance providers for dalfampridine following prescription by the neurologist (*n* = 2). Since this was a proof-of-concept pilot study with the principal aim to explore effect sizes of combined dalfampridine and physical therapy compared to physical therapy alone, we only analyzed data for subjects who completed the full intervention protocol (*n* = 4 each cohort).

Four of the 8 completers had been prescribed dalfampridine by their physician, and 4 were not taking the medication. Only 5 of the 8 participants who completed the intervention were available to attend the 1-month follow-up; the 3 who missed their follow-up assessments were due to travel/holidays. Thus, the 1-month follow-up timepoint was omitted from the analyses reported herein.

### Interventions

Participants taking dalfampridine took 10 mg doses every 12 h per physician prescription. The physical therapy intervention has been described elsewhere (11) but critical components are detailed here.

The physical therapy intervention was a progressive, mobility and balance motor-relearning (i.e., restorative-focused) intervention provided one-on-one by a licensed physical therapist that included training in functional strengthening, coordination, static and dynamic balance, dual-tasking, and gait. The intervention was standardized in terms of the philosophical approach and structure, but the specific activities were customized to the participant's individual needs (selected from a range of defined activities within the components of the program) and difficulty level. This intervention model is consistent with current clinical practice, which is characterized by a multimodal approach (12). Moreover, this intervention is based on the current best evidence demonstrating that multimodal interventions produce larger improvements in mobility outcomes in people with MS than unimodal interventions (13).

Physical therapy was provided at our research facility or clinical practice location two times per week for 6 weeks (12 sessions). Each session included 40 min of therapeutic intervention, lasting approximately one hour in total. Using theoretical frameworks for motor relearning, each session comprised three specific components: (i) part-practice (10 min), (ii) whole-practice (20 min), and (iii) contextual practice (10 min) to facilitate transfer to real world environments. Additionally, home practice was encouraged but not tracked and relevant patient education was provided.

Training activities were performed initially in closed environments (quiet room) and progressed on an individual basis to open environments (e.g., busy corridor area; background conversations). An intervention catalog in the Manual of Procedures listed the specific activities for each training component as well as five prescribed levels of difficulty for training progression. Therapists had autonomy in the selection of activities to address individual-specific impairments and customization of difficulty to ensure that a high degree of challenge was maintained throughout. Exercise programs that provide a high degree of challenge are consistently more effective than those providing only a moderate degree of challenge (14). This personalized approach, while adhering to the structured principles and components of this motor-relearning intervention, was considered to maximize potential for individual therapeutic gain while simultaneously ensuring that the target intensity for each activity could be achieved. Treatment fidelity was ensured by having all therapists undergo a 3-h training in the intervention protocol, co-treating with the first author for at least 3 sessions, providing a detailed Manual of Procedures to guide clinical decision-making, and periodic auditing of the intervention sessions and documentation. Three therapists, including the first author, provided the intervention.

For **part-practice**, therapists selected activities that targeted static and dynamic standing balance, lower extremity coordination, functional strengthening, and single-step training (e.g., swing and stance control). Intensity of part-practice was documented as sets and repetitions of each activity, with the goal to complete 2–3 sets of 12–15 repetitions of 2 different activities in 10 min. **Whole-practice** included activities that involved continuous practice of gait. Whole-task practice was a mixture of overground gait training and treadmill gait training (no body weight support). Whole-task activities also included narrow walking, side-stepping, backward walking, and speed modulation. For treadmill walking, participants were encouraged *not* to hold onto the handrail to maximize practice with full weight bearing and to improve dynamic balance and confidence, as well as maximize degree of challenge. Therapists provided intermittent assistance at the trunk or lower limbs as needed to facilitate balance and kinematics of limb movement. Emphasis was on motor control, not aerobic training, although speed was increased when possible. Intensity of whole-practice was documented by continuous minutes and bouts of treadmill walking, and sets and repetitions of overground practice activities. If a session included overground practice only, then at least 2 activities with 2 sets of 12–15 repetitions was performed in 20 min.

**Contextual transfer practice** included obstacle negotiation, stair climbing, stopping and turning, terrain/surface/lighting changes, and outdoor walking. Contextual transfer practice was an extension of whole-practice that applied key motor-learning principles of task variability, progression, and challenge, and was always conducted overground in the “real world” (e.g., hallways, cafeteria, escalators, and outdoors pending weather). Two contextual transfer activities were performed in each session. Since these activities involved real-world practice of continuous ambulation in various contexts, intensity of contextual practice was measured by number and duration of rests required to obtain 10 min of practice. Dual-task training was incorporated as part of the intervention (starting in week 2 of the program, as appropriate) to increase the challenge and provide task-specific practice of ecologically-valid mobility tasks (e.g., talking while walking). During dual-task training, participants performed cognitive tasks while practicing gait and balance activities. Several different cognitive activities were used with two different activities assigned to each session to ensure all participants practiced a range of dual-tasks (11).

As is customary in outpatient physical therapy, home-based practice of the skills (part and/or whole) was asked of the participants to enhance their ability to transfer and consolidate learning to everyday mobility situations. It was assumed that contextual transfer practice was occurring during everyday mobility activity in the home and community environments. Patient education included advice on stretching, fatigue/energy management, and strategies for transitioning to ongoing home and community practice after the completion of the 6-week intervention.

### Outcome Measures

The primary outcome was T25FW (timed using a handheld stopwatch) after the physical therapy/combined intervention. The T25FW was used primarily to enable direct comparison with the dalfampridine clinical trials. We also measured self-selected single-task and dual-task gait speed. For the dual-task, participants walked at their self-selected speed while performing the auditory “clock task” (15). Response time and accuracy were recorded during walking (dual-task) and during seated performance of the clock task (single-task). Gait data were acquired using a 20-foot instrumented walkway (ProtoKinetics, Havertown, PA). For both single-task and dual-task walking, participants completed 4 continuous passes across the walkway with turns performed off the walkway such that only steady state strides were used in the analysis.

Secondary outcome measures were selected to explore potential treatment effects on performance-based and patient-reported measures of balance, cognition, and fatigue. These included the Mini-BESTest, Four-Square Step Test, the Symbol-Digit Modalities Test, and self-reported outcomes for walking disability (12-Item MS Walking scale; MSWS-12), fatigue (Fatigue Severity Scale), balance self-efficacy (Activities-specific Balance Confidence scale), and quality of life (MS Impact Scale, MSIS-29).

The outcome measures were administered at (i) initial baseline, which was before dalfampridine was commenced in the medication group (Week 0), (ii) after the 3-week run period, which was the drug-only phase for the D+PT group and no-treatment phase for the PT group (Week 3), (iii) after the 6-week physical therapy intervention (Week 9), and (iv) 1-month after completion of the 6-week intervention. Since only 5 of the 8 participants were available to complete the follow-up visit at their scheduled time, these data were omitted from the statistical analyses. Outcome assessments were conducted by a trained evaluator who was naïve to dalfampridine status of each participant at each timepoint.

### Data Analysis

The groups were compared at baseline using independent samples *t*-tests or non-parametric tests as appropriate. Due to the preliminary nature of this pilot study, we placed most emphasis on the effect size comparison and confidence intervals rather than statistical significance. Thus, the main analysis was a between-group comparison of absolute change scores during the physical therapy phase (i.e., change between Week 3 and Week 9 for D+PT vs. PT). Effect sizes are reported as Cohen's *d*. Given the small and exploratory nature of the study, we also observed individual response patterns in the primary outcome measure, T25FW.

## Results

The characteristics of the participants are presented in Table 1. At Week 0 (study enrollment), the two groups did not differ significantly in age, disease duration, education, Symbol-Digit Modalities Test, Mini-BESTest, Four Square Step Test, T25FW, fatigue, self-selected gait speed, or dual-task gait speed. However, the D+PT group, on average, was relatively more impaired than the PT groups in most outcomes at baseline, largely driven by one outlier with EDSS 6.5 (Table 1). The D+PT group had significantly higher self-rated walking disability (*p* = 0.029) and lower balance self-efficacy at Week 0 (*p* = 0.046), as well as slightly higher median EDSS and a higher median number of falls in the last 12 months, but these latter differences were not statistically significant.

**Table 1:** Demographic and baseline characteristics of participants in each group.

**Participant**	**Age (years)**	**Sex**	**Years since diagnosis**	**Type of MS**	**Falls in last year**	**EDSS**	**T25FW (s)**	**STGS (m/s)**	**DTGS (m/s)**	**MSWS-12 (transformed score)**	**Assistive device**
**Dalfampridine plus physical therapy (*****n =*** **4)**											
D1	59	F	6	RRMS	1	3.0	6.61	0.96	0.98	91.7	None
D2	59	F	12	RRMS	7	6.5	8.36	0.60	0.74	72.9	Rollator
D3	42	F	4.3	RRMS	2	6.0	9.07	0.64	0.65	87.5	Cane
D4	38	F	2	SPMS	12	6.5	13.86	0.49	0.30	89.6	Quad cane
Mean/Median	49.5		6.1		4.5	6.3	9.47	0.67	0.67	85.4	
(SD/IQR)	(11.1)		(4.3)		(1.3–10.8)	(3.8–6.5)	(3.1)	(0.20)	(0.28)	(8.5)	
**Physical therapy (*****n** **=*** **4)**											
P1	63	F	15.5	SPMS	1	4.5	6.36	1.09	0.95	47.9	None
P2	65	F	5.8	RRMS	1	3.0	6.27	1.07	1.19	50.0	None
P3	53	M	0.5	PPMS	3	6.0	12.84	0.54	0.41	83.3	Cane
P4	29	F	5	RRMS	2	6.0	6.0	0.98	0.72	54.2	Cane
Mean/Median	52.5		6.9		1.5	5.3	7.9	0.92	0.82	58.9	
(SD/IQR)	(16.5)		(6.3)		(1.0–2.8)	(3.4–6.0)	(3.3)	(0.26)	(0.33)	(16.5)	

*F, female; IQR, interquartile range; M, male; PPMS, primary progressive MS; RRMS, relapsing-remitting MS; SD, standard deviation; SPMS, secondary progressive MS; T25FW, Timed 25-foot walk (seconds); STGS, single-task gait speed (meters per second); DTGS, dual-task gait speed (meters per second); MSWS-12, 12-Item MS Walking Scale*.

### Timed 25-Foot Walk

The T25FW data are presented in Table 2. During the 3-week drug-only run-in phase (Week 0-3), the D+PT group decreased T25FW time from 9.5 s (SD 3.1 s) to 8.3 s (SD 3.2 s; *p* = 0.032, *d* = 1.89), which represented a 12.8% improvement (95% CI 1.2 to 24.4%). During the no-treatment phase (Week 0–3) for the PT group, T25FW also decreased from 7.9 s (SD 3.3 s) to 7.0 s (SD 2.8 s, *p* = 0.133; *d* = 1.02), which represented a 10.0% improvement (95% CI −4.6 to 24.5%); however, this appeared to be driven by one outlier (see Table 2, P4) who walked 1.3 s faster at the second baseline visit. With the outlier removed, the PT group change during Week 0–3 was −6.3% (95% CI to 22.8 to 10.2%).

**Table 2:** Timed 25-foot walk test data by subject and group (faster times are better; negative percentages are improvement).

**Participant**	**Week 0 T25FW (s)**	**Week 3 T25FW (s)**	**Week9 T25FW (s)**	**% change Week 0–3**	**% change Week 3–9**	**% change Week 0–9**
**Dalfampridine plus physical therapy (*****n =*** **4)**						
D1	6.61	6.14	5.24	−7.1%	−14.7%	−20.7%
D2	8.36	6.74	4.27	−19.4%	−36.7%	−48.9%
D3	9.07	7.36	6.19	−19.0%	−15.9%	−31.8%
D4	13.86	13.05	11.02	−5.8%	−15.6%	−20.5%
Mean	9.48	8.32	6.68	−12.8%	−20.7%	−30.5%
(SD)	(3.10)	(3.19)	(3.00)	(7.3%)	(10.7%)	(13.4%)
**Physical therapy (*****n =*** **4)**						
P1	6.36	6.24	5.81	−1.9%	−6.9%	−8.7%
P2	6.27	6.08	4.88	−3.0%	−19.8%	−22.3%
P3	12.84	11.05	10.27	−13.9%	−7.1%	−20.0%
P4	6.00	4.74	4.30	−21.0%	−9.4%	−28.4%
Mean	7.87	7.03	6.31	−10.0%	−10.8%	−19.8%
(SD)	(3.32)	(2.76)	(2.71)	(9.2%)	(6.1%)	(8.3%)

During the 6-week physical therapy intervention phase (Week 3–9), the D+PT group further decreased T25FW time to 6.7 s (SD 3.0 s; *p* = 0.021, *d* = 2.24), which represented a further 20.7% improvement (95% CI 3.8 to 37.6%) for an overall Week 0–9 improvement of 30.5% (95% CI 9.2 to 51.8%). The PT group further decreased T25FW time to 6.3 s (SD 2.7 s, *p* = 0.029, *d* = 1.96), which represented a further 10.8% improvement (95% CI 1.0 to 20.5%) for an overall Week 0–9 improvement of 19.8% (95% CI 6.7 to 33.0%).

The between-group comparison of the absolute change in T25FW time between Week 3 and Week 9 favored the D+PT group but was not statistically significant (mean difference [MD] = −0.93s, 95% CI −1.9 to 0.07s, *p* = 0.064, *d* = 1.6). The results were similar when comparing the groups on percent change in T25FW between Week 3 and Week 9: there was a nonsignificant but large effect in favor of the D+PT group (MD = 9.9%, 95% CI −5.1 to 24.9%, *d* = 1.1).

Among the 4 participants taking dalfampridine, none of the participants achieved 25% improvement in T25FW on 3 weeks of the drug alone, which was the average improvement observed in dalfampridine clinical trial “responders” (3). After adding 6 weeks of physical therapy, all 4 participants taking dalfampridine demonstrated T25FW improvements >20% from baseline, with 2 participants exceeding 30% improvement (Table 1). The relative improvements in T25FW among the PT-only group in response to the physical therapy intervention were generally smaller, ranging from 6.9 to 19.8%.

### Self-Selected Single and Dual-Task Gait Speed

**Figure 1:**
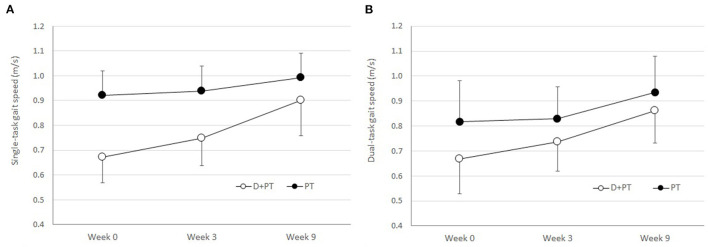
**(A)** Single-task gait speed and **(B)** dual-task gait speed by group and time point. Week 0–3 represents dalfampridine only for D+PT group and no-treatment phase for PT group. Week 3–9 represents the physical therapy phase with or without dalfampridine. D+PT denotes dalfampridine plus physical therapy; PT denotes physical therapy without dalfampridine. Error bars are SEM.

The improvement in self-selected single-task gait speed during the physical therapy intervention phase (Week 3–9) favored the D+PT group, but it was not statistically significant (MD = 0.12 m/s, 95% CI −0.01 to 0.24 m/s, *p* = 0.070, *d* = 1.56). There were similar improvements in dual-task gait speed in both groups (MD = 0.02 m/s, 95% CI −0.11 to 0.14 m/s, *p* = 0.747, *d* = 0.24), illustrated in Figure 1.

There were no significant changes in dual-task effects on gait speed or clock-task performance across time or between groups. Dual-task performance was characterized by large between-subject variability in both gait speed and the clock task.

### Secondary Outcome Measures

The secondary outcome measures are presented in Table 3. The between-group intervention effect sizes for the physical therapy phase were moderate to large and favored the D+PT group for balance (Mini-BESTest), self-rated walking disability (MSWS-12), and balance self-efficacy (ABC), but only the ABC was statistically significant. The D+PT group also had a slightly greater improvement on the MSIS-29, but the between-group effect size was small. The PT group had a significantly greater improvement on the Symbol-Digit Modalities Test than the D+PT group. The Four Square Step Test showed slight worsening (increase in time) after the intervention in both groups, but slightly more so in the D+PT group (small effect size). There was no remarkable change in fatigue for either group despite moderately severe fatigue at baseline.

**Table 3:** Mean (SD) for secondary outcome measures and between-group differences in change during physical therapy (PT) intervention phase (Week 3–9).

	**Dalfampridine + Physical therapy (*****n =*** **4)**						**Physical therapy (*****n =*** **4)**						**Between-group difference in PT phase change (Week 9 minus Week 3) (D+PT minus PT)**	
	**Week 0**		**Week 3**		**Week 9**		**Week 0**		**Week 3**		**Week 9**		**MD (95% CI)**	***d***
Mini-BESTest (max. 28)	13.5	(7.6)	15.8	(7.5)	19.8	(5.1)	21.8	(3.3)	22.0	(2.8)	22.5	(4.8)	3.5 (−7.2, 14.2)	0.68
Four-Square Step Test (s)	20.3	(7.9)	17.2	(7.5)	19.4	(10.9)	15.9	(8.4)	13.6	(5.1)	14.2	(9.1)	1.6 (−5.2, 8.3)	0.41
SDMT (number correct)	47.8	(9.9)	50.8	(10.4)	49.8	(8.5)	50.8	(7.9)	50.5	(8.3)	55.5	(6.6)	−6.0 (−10.1, −1.9)	2.55
MSWS-12 (0–100 transformed)	85.4	(8.5)	68.2	(18.3)	44.3	(10.0)	58.9	(16.5)	53.6	(17.9)	48.9	(23.5)	−19.3 (−42.9, 25.8)	1.41
ABC (%)	47.0	(17.5)	45.2	(13.8)	66.7	(18.4)	73.8	(12.2)	69.4	(18.0)	76.4	(15.1)	14.5 (7.1, 22.0)	3.39
Fatigue Severity Scale (max. 64)	55.0	(14.0)	45.5	(23.3)	43.3	(16.3)	40.8	(8.7)	45.8	(9.4)	43.0	(18.1)	0.5 (−14.3, 15.3)	0.06
MSIS-29 (max. 145)	91.0	(19.8)	74.8	(13.9)	67.0	(17.5)	70.8	(15.9)	69.5	(18.6)	70.5	(18.2)	−8.8 (−43.3, 25.8)	0.44

*ABC, Activities-specific Balance Confidence scale; MD, mean difference; MSIS-29, 29-Item Multiple-Sclerosis Impact Scale; MSWS-12, 12-Item Multiple Sclerosis Walking Scale; PT, physical therapy; SDMT, Symbol-Digit Modalities Test*.

## Discussion

The purpose of this pilot study was to demonstrate proof-of-concept of combining physical therapy with prescription dalfampridine to improve walking speed in people with MS. The effects of dalfampridine (alone) on walking speed have been well-established in several large clinical trials (1–3, 16, 17), but the proportion of people who experience a meaningful response to the drug is fewer than 40% (3). Physical therapy is the other mainstream treatment approach for mobility limitations in people with MS, but the effect of combining these two interventions has not yet been systematically studied. The physical therapy intervention represented an evidence-based motor relearning approach that is consistent with outpatient clinical practice. This rehabilitation approach, despite being relatively conventional, is also not well studied in people with MS and has unknown responder rates. Thus, the control group in this study (PT only) provided preliminary effect estimates of this rehabilitation approach.

While we found that both groups improved walking speed in response to this physical therapy intervention, the relative improvement was almost twice as large in the group taking dalfampridine. Further, whereas none of the individuals taking dalfampridine met Hobart's criterion of “responders” (≥20% improvement in T25FW) (18) in response to dalfampridine alone, after 6-weeks of physical therapy concurrently with dalfampridine, all participants achieved >20% improvement from initial baseline. Thus, it is reasonable to assert that combining physical therapy with dalfampridine when the medication is first prescribed could improve the responder rate well above the previously observed 38%. It is surprising that physical therapy is not routinely prescribed with dalfampridine. The LIBERATE Trial, a post-authorization investigation of dalfampridine in a routine practice setting, found that only 14% of individuals prescribed dalfampridine received concurrent physical therapy (19). However, it is not clear whether dalfampridine is a first-line treatment choice by physicians for mobility impairments, or whether it is prescribed when physical therapy has failed or is declined by the patient.

The evidence for physical therapy in MS is presently limited by small studies and highly variable treatment protocols. Furthermore, rehabilitation research in MS has been dominated by “exercise training,” mostly comprising aerobic and resistance training (13, 20–23) and specialized, unimodal interventions such as robotic-assisted gait training (8, 9, 24) and body-weight supported treadmill training (5, 10, 25–27). There have been only a handful of studies that have examined a pragmatic intervention that is representative of physical therapy practice for neurological rehabilitation (28–30). This pilot study has demonstrated that the rigorous and progressive motor relearning intervention customized to individual ability can produce important improvements in patients with mild to moderate mobility limitations.

The D+PT group had significantly greater improvement in the MSWS-12, reflecting improved self-perceived walking ability, with the average improvement during the PT period alone being 23.9 points, far exceeding the minimally important change of 10.4 points (31). The finding that the PT group did not report meaningful improvement in self-rated walking disability is likely due to starting with only moderate self-perceived walking disability at baseline, compared to the very high disability rating of the D+PT group. Further, the PT group had mean T25FW under 8 s at baseline, thus creating a potential ceiling effect. Nonetheless, 2 of the 4 participants in the PT-only group had MSWS-12 improvements exceeding 10.4 points, while one of the fastest walkers at baseline reported no change, and one participant reported 8.8 point decline. Interestingly, the latter participant also reported increased fatigue at Week 9, which may have influenced her walking disability perception. It is also noteworthy that even though the D+PT group were “non-responders” to dalfampridine (alone) using Hobart's criterion of ≥20% improvement on T25FW (18), the average patient-perceived improvement on the MSWS-12 during the drug-only run in was an astonishing 17.2 points. Thus, it may be necessary to also consider patient perception of improvement when defining responders, rather than relying solely on objective measures of (fast) gait speed. Indeed, it has been suggested that T25FW in combination with MSWS-12 may be optimal for determining response to dalfampridine (32).

It was unexpected that the PT group, on average, experienced an improvement in T25FW time during the no-treatment baseline phase. However, this change was largely driven by one individual, the fastest walker at baseline, who walked 21% faster at the second baseline visit. It is not uncommon to observe small systematic increases in gait speed between multiple baseline assessments in individuals with mobility disability (33), which could be due to greater comfort level with testing procedures and environment on repeat occasions. Importantly, this magnitude of variation during the no-treatment phase was not observed for self-selected gait speed in either single-task or dual-task conditions. Thus, we believe the observed effects on the T25FW during the no-treatment phase for the PT group are likely due to the T25FW being a test of fastest gait speed, which could be influenced by how the instructions for the test were delivered or emphasized on each occasion, as well as personal factors such as fatigue or motivation on any given day.

The fact that dual-task gait speed improved equivalently in both groups is quite encouraging. This finding suggests that our physical therapy program was able to improve gait automaticity regardless of dalfampridine treatment. We can infer improved gait automaticity from the physical therapy intervention since there was no reciprocal decline in the cognitive task performance associated with the improvement in dual-task gait speed (34). Although there was no between-group difference in change on the Mini-BESTest scores (range 0–28, higher scores indicate better balance), the D+PT group demonstrated an average improvement during the D+PT phase of 4 points (and a 6.3 point increase overall), which is considered clinically important (35). The therapy-related change in the Mini-BESTest was smaller and likely not meaningful in the PT group (<2 points on average), but this could be due to a higher initial baseline score. Perhaps more important to note is that the two most severely impaired individuals (both in the D+PT group) with initial Mini-BESTest scores of 6 and 11, respectively, each improved by only one point on the drug alone, but by 7 and 12 points, respectively, with PT. Whether gains this size among severely impaired individuals could be achieved with this PT program without concurrent dalfampridine treatment is unclear; there were no participants in the PT group with equivalent balance impairment at baseline. The Four Square Step Test showed slight worsening after the intervention in both groups, but we believe this could reflect greater caution as opposed to worse balance, especially when considered alongside the Mini-BESTest results.

There are several limitations that must be acknowledged. Inarguably, the small sample size is a limitation. However, the study achieved its purpose in demonstrating proof-of-concept and obtaining effect size estimates for PT with and without dalfampridine. Although our point estimates lack precision, the group results (many of which were statistically significant) together with visual analysis of the individual patterns and the large effect sizes point to the value of further, larger investigations. The between-group comparisons on walking speed outcomes are limited by the non-randomized design. The non-randomized design was necessary because the budget did not enable the investigators to provide the medication as part of the study. Consequently, the groups were not directly comparable on disability at baseline. The tendency for higher disability in the D+PT group is not surprising and may have contributed to the reasons these individuals were prescribed dalfampridine clinically. Future study designs wishing to examine therapeutic efficacy in patients routinely prescribed dalfampridine should endeavor to match control participants on disability level at baseline or consider a randomized design with placebo medication. Because we recruited patients who were prescribed dalfampridine as part of their routine clinical care, we relied on physician referral to the study, which posed some degree of challenge for recruitment. Volunteers for the PT-only group self-referred to the study via community advertisements. Matching PT-only participants to the D+PT group would have further delayed study enrollment. The physical therapy intervention in this study was limited to 6 weeks (12 sessions), which approximated the typical outpatient physical therapy practice for patients with MS in our hospital system at the time of the study. The study is lacking follow-up analysis. However, the objective of this study was to assess immediate effects of PT with and without dalfampridine on gait speed and related outcomes, to assess whether future investigations would be worthwhile.

## Conclusion

The findings from this proof-of-concept pilot study provide promising new evidence that physical therapy that adheres to motor relearning principles and the challenge framework, provided concurrently with dalfampridine, may offer potential benefit to patients with MS who fail to achieve meaningful improvement after treatment with dalfampridine alone. Dalfampridine combined with physical therapy is worthy of further, controlled investigation.

## Data Availability Statement

The raw data supporting the conclusions of this article will be made available by the authors, without undue reservation.

## Ethics Statement

The studies involving human participants were reviewed and approved by University of North Carolina at Chapel Hill. The patients/participants provided their written informed consent to participate in this study.

## Author Contributions

PP conceptualized and designed the study, with input from BG and SM-P. PP developed and delivered the physical therapy intervention, trained and supervised project staff, collected data, conducted the data analysis, and lead the writing of the manuscript. PP and BG acquired the funding. PP and SM-P recruited participants. All authors contributed to the revision, editing, final development of the manuscript, and presentation of data.

## Funding

This pilot study was funded by the National Multiple Sclerosis Society (PP-1503-03495).

## Conflict of Interest

PP has served as a consultant and member of the Physical Therapy in Post Ischemic Stroke Advisory Board for Acorda Therapeutics, Inc. The remaining authors declare that the research was conducted in the absence of any commercial or financial relationships that could be construed as a potential conflict of interest.

## Publisher's Note

All claims expressed in this article are solely those of the authors and do not necessarily represent those of their affiliated organizations, or those of the publisher, the editors and the reviewers. Any product that may be evaluated in this article, or claim that may be made by its manufacturer, is not guaranteed or endorsed by the publisher.
